# Effect of the Number of Pregnancies on Mortality Risk in HIV-Infected Women: a Prospective Cohort Study in Rural KwaZulu-Natal, South Africa

**DOI:** 10.1007/s10461-018-2232-0

**Published:** 2018-08-02

**Authors:** Hyunsuk Yoo, Juyeon Lee, Jae-Joon Yim, Till Bärnighausen, Frank Tanser, Sue K. Park

**Affiliations:** 1 Department of Medicine, Seoul National University College of Medicine, Seoul, South Korea; 2 Department of Preventive Medicine, Seoul National University College of Medicine, 103 Daehakno, Jongro-Gu, Seoul, 110-799 South Korea; 3 Department of Biomedical Science, Seoul National University College of Medicine, Seoul, South Korea; 4 Division of Pulmonary and Critical Care Medicine, Department of Internal Medicine, Seoul National University College of Medicine, Seoul, South Korea; 5 Department of Global Health and Population, Harvard School of Public Health, Boston, MA USA; 6 Cancer Research Institute, Seoul National University, Seoul, South Korea; 7 Africa Health Research Institute, KwaZulu-Natal, South Africa; 8 School of Nursing and Public Health, University of KwaZulu-Natal, Durban, South Africa; 9 Centre for the AIDS Programme of Research in South Africa (CAPRISA), University of KwaZulu-Natal, Durban, South Africa; 10 Research Department of Infection & Population Health, University College London, London, UK; 11 Heidelberg Institute of Global Health, Faculty of Medicine, Heidelberg University, Heidelberg, Germany; 12 Institute of Global Health, University College London, London, UK

**Keywords:** HIV, Pregnancy, Tuberculosis, AIDS, Mortality

## Abstract

We investigated whether mortality risk increases with the number of full-term pregnancies in HIV-infected women. Our study is based on data from the ACDIS cohort, collected in rural KwaZulu-Natal, South Africa. Mortality risk for different number of pregnancies in HIV-infected women was analyzed using Cox proportional hazards model. The risk of TB or AIDS mortality in HIV-uninfected women did not change with the number of full-term pregnancies, while the corresponding risk increased markedly in HIV-infected women. The risk of TB or AIDS mortality increased 1.48-fold (95% CI 1.25–1.75), 1.76-fold (95% CI 1.45–2.13), and 1.59-fold (95% CI 1.31–1.94) for one, two, and three or more full-term pregnancies compared to none, respectively. Finally, women who are young (age < 26) have greater risk of TB or AIDS mortality compared to women who are old (age ≥ 26), and women residing in rural areas have greater risk compared to women who reside in non-rural areas.

## Introduction

Human immunodeficiency virus (HIV) is a leading cause of mortality in rural South Africa, where HIV infection accounts for up to 61 and 73% deaths among male and female young adults, respectively [1]. Many of the health complications associated with HIV infection are due to the loss of immunity that arises during the course of the disease, which leads to opportunistic infections that define AIDS: tuberculosis (TB), candidiasis, invasive cervical cancer, cryptococcosis, Kaposi’s sarcoma, and many other diseases [2–4]. Among these opportunistic infections, TB remains one of the most common, and the most serious infections in South Africa [5]. South Africa, with just 0.7% of the world’s population, has one of the world’s worst TB epidemics, compounded by multi-drug resistant TB (MDR-TB) and HIV co-infection [6].

The influence of pregnancy on the risk of mortality in HIV-infected women is complex. Meta-analyses of various studies conducted in Sub-Saharan Africa have shown that the risk of mortality during pregnancy and the postpartum period among HIV-infected pregnant women is 3.7–21.6 times that of uninfected pregnant women [2, 7, 8]. In particular, in regions where HIV prevalence is greater than 15%, more than 50% of maternal mortality was attributable to HIV [2]. However, in contrast to previous studies, one study reported the paradoxical effect of pregnancy on the risk of mortality. According to a study published in 2013, the excess mortality attributable to HIV in non-pregnant women (51.8 per 1000 person-years) was higher than that in pregnant or post-partum women (11.8 per 1000 person-years) [9]. The authors suggested that as only healthy women get pregnant, pregnancy may appear to reduce mortality associated with HIV. Thus, the results were distorted owing to “a healthy pregnant women effect” [9].

As pregnancy can accelerate the progression of HIV disease we hypothesized that pregnancy increases the risk of mortality from complications of AIDS in HIV-infected women and that the risk of mortality increases with the number of pregnancies [3]. Until now, only a few studies have examined the effect of pregnancy on long-term risk of mortality due to HIV and, to our knowledge, there have been no studies that have investigated the effect of the number of pregnancies on HIV-related mortality in HIV-infected women [2, 9]. We are the first to investigate the influence of the number of pregnancies on the long-term risk of mortality due to HIV-related infections in a population-based cohort.

The aim of this study was to compare the risk of mortality with the number of pregnancies in HIV-infected women and HIV-uninfected women based on the Africa Health Research Institute (AHRI) (previously Africa Centre for Health and Population Studies) population-based cohort data collected from rural KwaZulu-Natal, South Africa.

## Methods

### Study Design and Cohort Description

This was a prospective cohort study based on data from the ACDIS cohort, collected between 2000 and 2013 in KwaZulu-Natal, South Africa.

The ACDIS data were provided by AHRI. The ACDIS dataset contains longitudinal records for individuals residing in the Umkanyakude district, KwaZulu-Natal, who were surveyed between January 2000 and June 2013 [10]. The prevalence of HIV in this cohort was very high, with 29% of residents HIV-infected in 2011 [11]. The crude HIV incidence among residents was estimated to be 2.63 infections per 100 person-years (95% CI 2.50–2.77), with a cumulative HIV infection rate of 74% for female residents by the age of 49 years [12].

The ACDIS data were collected since January 2000 and were based on 12,000 households containing 90,000 household members in the predominantly rural, Zulu-speaking, 438 km^2^ demographic area of Mtubatuba [13]. To fulfil the eligibility criteria for the ACDIS cohort, individuals must be a member of a household within the surveillance area but not necessarily be resident within it [10]. The homesteads in rural areas are mostly scattered and the households are multi-generational, with an average size of 7.9 (standard deviation [SD] = 4.7) members [10]. Although predominantly rural, the area also contains an urban township and informal peri-urban settlements, which are typical of many other rural areas of South Africa [10]. In 2013, the concluding year of the cohort, the ACIDS cohort included 197,537 individuals aged ≥ 15 years.

The ACDIS data were collected semi-annually at a household level, and annually at an individual level. Household level data included pregnancy outcomes (abortions, and still and live births), mortality, migration of household residents, as well as socio-economic data (education and employment status). If the household members died during the observation period, the cause of death was recorded. Individual level data included HIV status and sexual behavior, including pregnancy history [10]. The “number of full-term pregnancies” was defined as the number of times a woman gave birth to one or more live-born children. “First interview” was defined as the episode in which first data collection of a participant was made, while “last interview” was defined as the episode in which last data collection was made. The total episodes of information collection ranged from 1 to 34 for different women. In order to diagnose HIV, field workers collected blood by conducting finger prick tests and sequentially the blood was tested for HIV according to the Joint United Nations Programme on HIV/AIDS (UNAIDS) and World Health Organization (WHO) guidelines [14]. Antibody testing was conducted with a broad-based HIV-1/HIV-2 ELISA (Vironostika^®^ HIV-1 Microelisa System (Biomérieux, Durham, NC, USA) followed by a confirmatory ELISA (Wellcozyme HIV 1 + 2 GACELISA; Murex Diagnostics Benelux B.V., Breukelen, The Netherlands) [10].

### Study Population

**Fig. 1:**
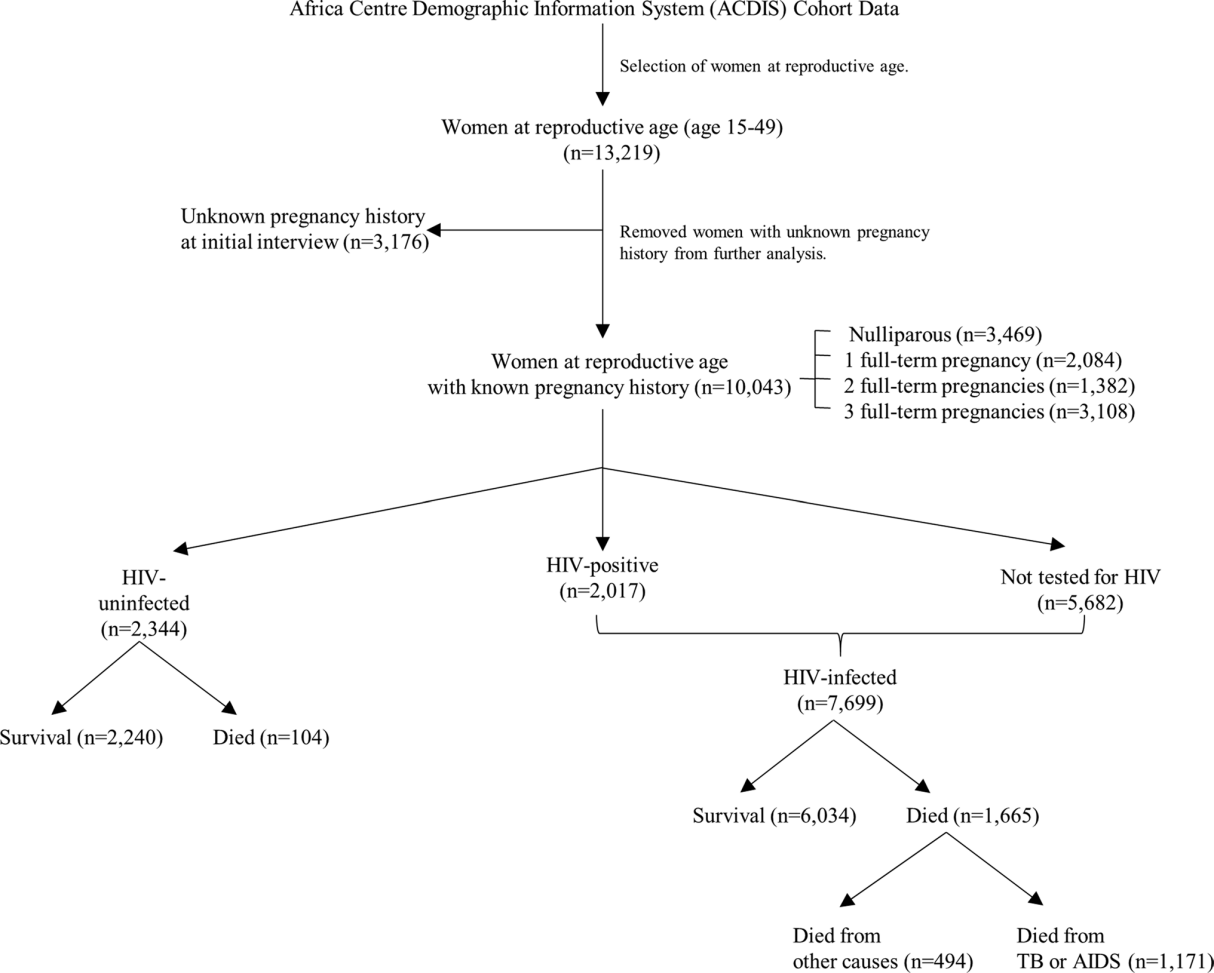
Study profile of selection of participants to assess the association between the number of full-term pregnancies and the risk of mortality from TB or AIDS and all-causes. *ACIDS* Africa Centre Demographic Information System, *AIDS* acquired immune deficiency syndrome, *HIV* human immunodeficiency virus, *TB* tuberculosis

Only women of reproductive age (15–49 years) at initial data collection were included in our analysis; women with an unknown pregnancy history were excluded from the analysis. The remaining with a known pregnancy history were divided into three groups based on their HIV status at the end of the study period: HIV-negative, HIV-positive, and not tested for HIV (Fig. 1).

In our study, HIV-positive women and those who did not test for HIV were defined as “HIV-infected women” based on the following reasons. TB or AIDS mortality rates were higher in HIV-infected women (9.5 per 1000 person-years) and women not tested for HIV (22.3 per 1000 person-years), as compared to only 0.8 per 1.000 person-years in HIV-negative women. Additionally, we noted that the proportion of TB or AIDS deaths in women not tested for HIV (70%) was similar to that of HIV-infected women (72%), and was significantly different from that of HIV-uninfected patients (24%) (Table 1). Finally, evidence from existing studies show that the proportion of HIV-infected adults who were undiagnosed was as high as 80% in the early 2000s [15]. Moreover, according to National Health Act 61 of 2003, Section 14, of South Africa, all clients have the right to refuse HIV testing without compromising their access to standard healthcare. This ACDIS study also did not require mandatory HIV testing; all tests were voluntary and informed consent was obtained from all participants. Therefore, women not tested for HIV were combined with HIV-infected women for further analysis.

**Table 1:** All-cause and TB or AIDS mortality rates for women with different numbers of full-term pregnancy, categorized based on HIV status

Full-term pregnancy	Person-years	Death from TB or AIDS, N	Mortality rate (/1000 PY)	Person-years	All-cause death, N	Mortality rate (/1000 PY)	Proportion of deaths from TB or AIDS^a^ (%)
HIV-uninfected, total	30,895	25	0.8	31,663	104	3.3	24
0	8927	6	0.7	9058	21	2.3	29
1	3581	5	1.4	3651	12	3.3	42
2	2969	0	0.0	3020	5	1.7	0
3+	15,418	14	0.9	15,934	66	4.1	21
HIV-infected, total	25,277	239	9.5	26,191	334	12.8	72
0	7765	50	6.3	7920	68	8.6	74
1	6333	56	8.6	6516	76	11.7	74
2	3633	29	7.5	3864	52	13.5	56
3+	7546	104	13.2	7891	138	17.5	75
Not tested for HIV, total	41,879	932	22.3	44,192	1331	31.1	70
0	17,045	238	13.4	17,749	351	19.8	68
1	9817	217	21.1	10,275	305	29.7	71
2	6040	199	30.8	6459	266	41.2	75
3+	8977	278	28.6	9709	409	42.1	68

Total number of all-cause deaths: 1769; total number of deaths from TB or AIDS: 1171

*AIDS* acquired immune deficiency syndrome, *HIV* human immunodeficiency virus, *TB* tuberculosis

^a^The proportion of TB or AIDS deaths was calculated as follows: [N of TB/AIDS deaths/N of all-cause deaths]

The number of full-term pregnancies for study participants were categorized as zero, one, two, and three or more. The cause of death in HIV-infected women was classified as mortality resulting from TB or AIDS or mortality from other causes.

Full ethical approval was received from the University of KwaZulu-Natal. The study population selection and analysis design were approved by the institutional review board (IRB) of Seoul National University Hospital (IRB number: 1607-098-776).

### Statistical Analysis

TB or AIDS mortality rates and all-cause mortality rates were calculated as the number of deaths per 1000 person-years and were stratified by HIV status. As the prevalence of TB in rural South Africa is very high, and the majority of HIV-infected individuals die from TB during HIV progression, deaths from TB, as well as AIDS, were categorized as HIV-related deaths [5].

Selected characteristics from HIV-infected women were compared between nulliparous and ever parous women and the differences in the characteristics were tested using student’s t-tests for continuous variables (age) and Chi square tests for categorical variables (marital status, area of residence, education level, and HIV status). We used the median age [26] of the HIV-infected women to stratify the data based on younger and older age groups. We defined a low education level as < 7 years of education because grades 1–6 are foundation and intermediate phases of the General Education and Training (GET), while grade 7 and higher are senior phases of the GET or Further Education and Training (FET) in South Africa. Continuous data were presented as means and SDs and categorical data were presented as frequencies and proportions.

A Cox proportional hazards model was used to examine the risk of mortality based on the number of pregnancies in HIV-infected women. Stratification analysis was additionally done to test how the effect of number of pregnancies on mortality risk changes according to age (< 26 vs. ≥ 26), area of residence (rural vs. non-rural such as peri-urban and urban and outside the surveillance area), and education level (unknown and low education level vs. high education level). P-heterogeneity tests were conducted to test for any significant differences in risk between strata group. P-interaction terms were also calculated to determine interactions between independent variables (age, area of residence, and education level) and number of pregnancies. To divide younger and older age groups, median age (26) of HIV-infected women was used. Low education level was defined as < 7 years of education because grades 1–6 are foundation and intermediate phases of the General Education and Training (GET), while grade 7 and higher are senior phases of the GET or Further Education and Training (FET) in South Africa.

The risk of death in each strata was expressed as HR and 95% CI and all HRs were adjusted HR values. Multi-collinearity between parity and confounding factors were examined using Pearson correlation coefficient and variance inflation factor (VIF) and the factors to be included in the multivariate analysis were selected. Factors included in the multivariate model were age (< 26 and ≥ 26), marital status (never been married, currently married, divorced or widowed, and unknown marital status), area of residence (rural, peri-urban, urban, and outside surveillance area and education level (< 1 year of education, 1–6 years of education, 7–12 years of education, ≥ 13 years of education, and unknown education level).

A spline plot was used to visualize the effect of the number of full-term pregnancies on risk of mortality from TB or AIDS and the risk of all-cause mortality in each HIV status group and HIV-infected women. *P*-values < 0.05 were considered statistically significant. All statistical analyses were conducted using R version 3.0.2 (R Foundation for Statistical Computing, Vienna, Austria) and SAS version 9.4 (SAS Institute, Cary NC, USA).

## Results

Overall, 13,219 women of reproductive age were identified; 3176 women with an unknown pregnancy history were sequentially excluded (Fig. 1). Thus in total, 10,043 fertile women with a known pregnancy history were enrolled in this study; 2344, 2017, and 5682 were HIV-uninfected, HIV-infected, and not tested for HIV, respectively (Fig. 1).

With an increasing number of full-term pregnancies in HIV-infected women (HIV-infected women and women not tested for HIV), both all-cause and TB or HIV-related mortality rates increased steadily. However, HIV-uninfected women had similar mortality rates regardless of the number of full-term pregnancies (Table 1).

There were total of 2787 nulliparous and 4912 ever parous women in the HIV-infected group (Table 2). The median age of HIV-infected women at initial interview was 26. Mean age was higher in the ever parous group (30.2 ± 8.1) than in the nulliparous group (21.2 ± 6.6). For both groups, there was highest proportion of women who were never married (74.2 and 53.1%), lived outside the surveillance area (44.6 and 37.6%), and had > 6 years of education (75.7 and 60.7%) at initial interview. In addition, HIV status at final episode was mostly not tested for HIV (78.6 and 71.1%, respectively) for both nulliparous and ever parous women. However, age, marital status, area of residence, and educational level at initial interview and HIV status at final episode for nulliparous and ever parous women were significantly different (*P* value < 0.05) (Table 2).

**Table 2:** General characteristics of HIV-infected women of reproductive age (15–49 years) selected from Africa Centre Demographic Information cohort, who were surveyed between January 2000 and June 2013

	Nulliparous (N = 2787)	Ever parous (N = 4912)	P-value^a^
	Mean (SD)	Mean (SD)	
Age at initial interview	21.2 (6.6)	30.2 (8.1)	< 0.01
	N (%)	N (%)	
Marital status at initial interview			
Never been married	2069 (74.2)	2606 (53.1)	< 0.01
Married, divorced or widowed	159 (5.7)	1409 (28.7)	
Unknown	559 (20.1)	897 (18.3)	
Area of residence at initial interview			
Rural	1034 (37.1)	1845 (37.6)	< 0.01
Peri-urban and urban	510 (18.3)	1222 (24.9)	
Outside surveillance area	1243 (44.6)	1845 (37.6)	
Educational level at initial interview			
Unknown or 1-6 year(s)	677 (24.3)	1932 (39.3)	< 0.01
> 6 years	2110 (75.7)	2980 (60.7)	
HIV status at final interview			
Infected	597 (21.4)	1420 (28.9)	< 0.01
Infection suspected but not tested	2190 (78.6)	3492 (71.1)	

HIV-infected women and women not tested for HIV were combined into the ‘HIV-infected group’ based on similar TB or AIDS mortality rates and proportions of TB or AIDS deaths (Table 1). Additionally, existing studies show that the proportion of HIV-infected adults who were undiagnosed was as high as 80% in the early 2000s [15]. Therefore, individuals not tested for HIV might have actually been HIV-infected

*HIV* human immunodeficiency virus, *SD* standard deviation

^a^For the continuous variable (age), student’s t-test was used. For categorical variables (marital status, area of residence, educational level and HIV status), Chi square tests were used

**Fig. 2:**
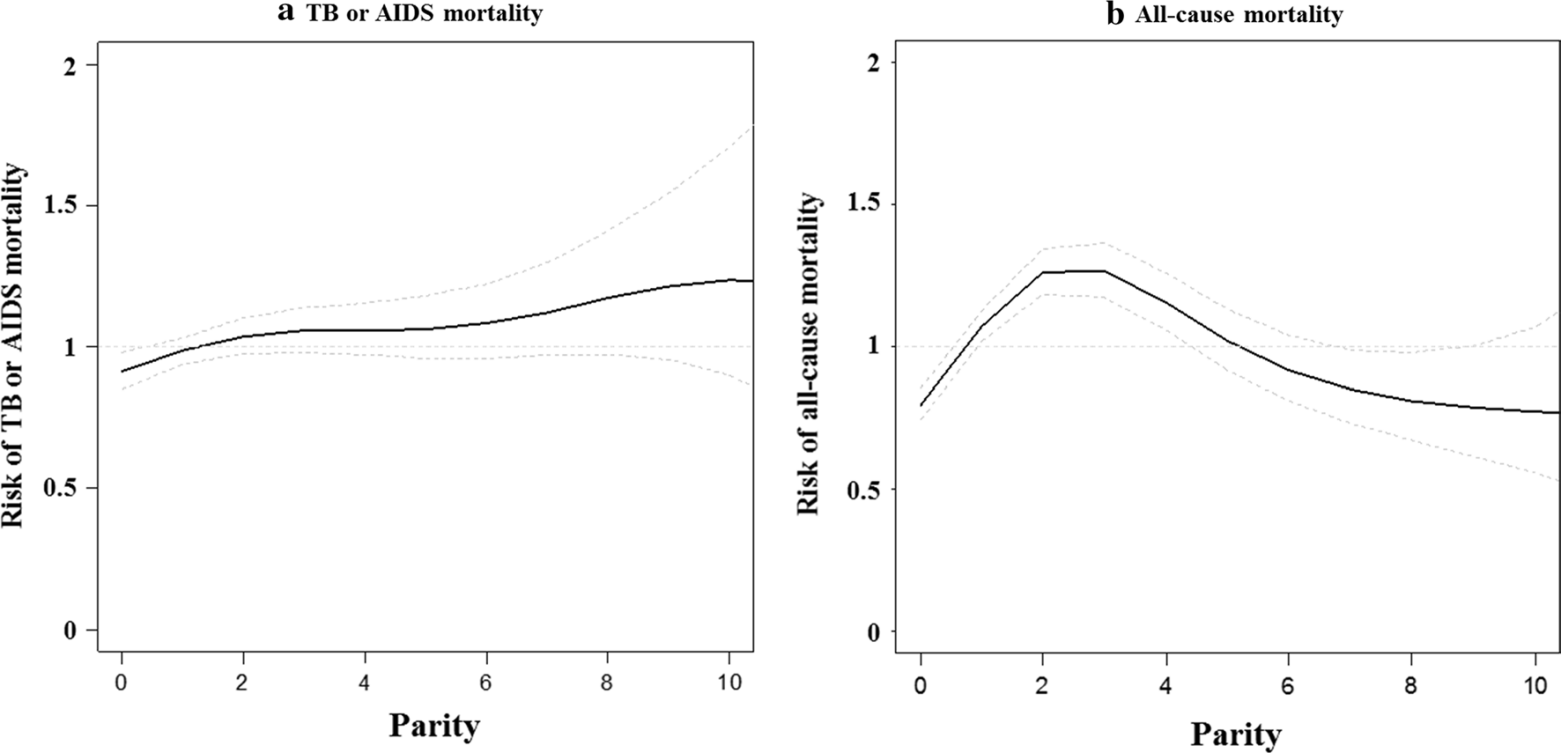
Spline plots visualizing the effect of parity on risk of mortality from **a** TB or AIDS and **b** all-causes in HIV-infected women. *AIDS* acquired immune deficiency syndrome, *HR* hazard ratio, *TB* tuberculosis

**Fig. 3:**
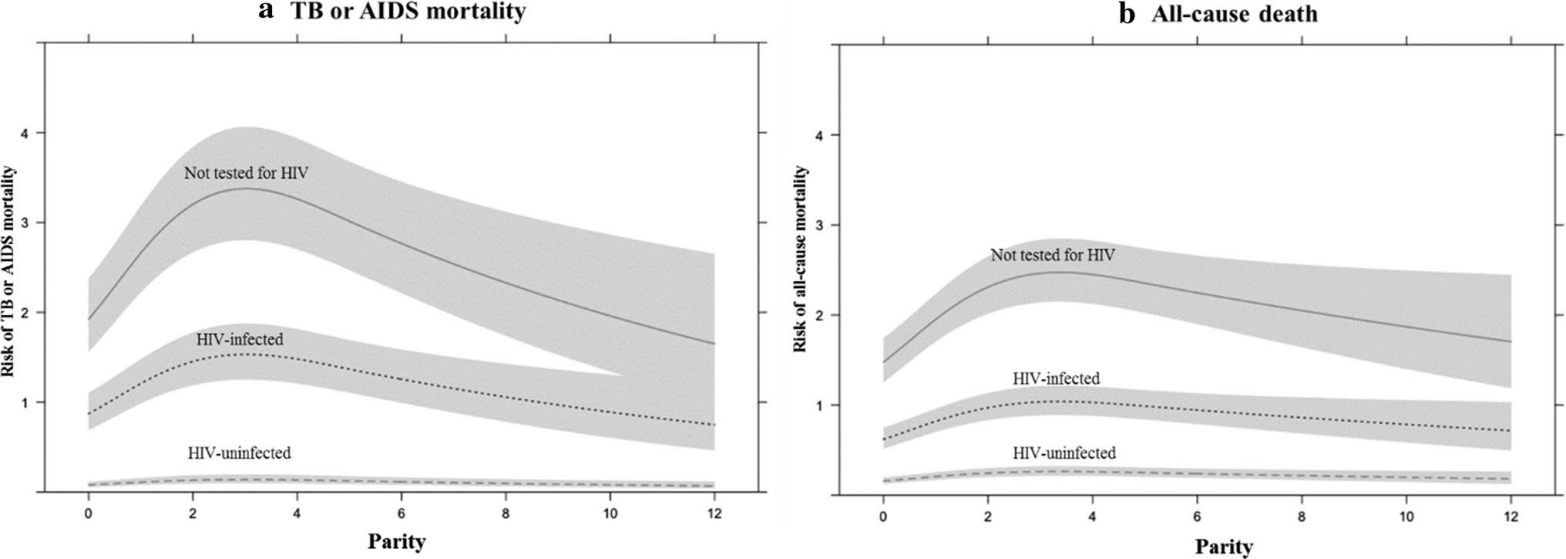
Spline plots visualizing the effect of parity on risk of mortality from **a** TB or AIDS and **b** all-causes in different HIV status group women of reproductive age. *AIDS* acquired immune deficiency syndrome, *HIV* human immunodeficiency virus, *TB* tuberculosis

The spline plot showed that the HR for all-cause mortality increased for up to two full-term pregnancies, plateaued near three full-term pregnancies, and then decreased for three or more full-term pregnancies, while the risk of mortality from TB or AIDS increased steadily with the increasing number of full-term pregnancies (Fig. 2). Both the risk of TB or AIDS mortality and all-cause mortality in HIV-uninfected women did not change with the number of full-term pregnancies (Fig. 3).

The risk of mortality from TB or AIDS increased by 1.48-fold (95% CI 1.25–1.75), 1.76-fold, and 1.59-fold for one, two, and three of more full-term pregnancies compared to no full-term pregnancy (Table 3). The risk of all-cause mortality was similar to the risk of mortality from TB or AIDS regardless of the number of pregnancies (Table 3).

**Table 3:** Effect of the number of full-term pregnancies on the risk of TB or AIDS and all-cause mortality in HIV-infected women of reproductive age

Full-term pregnancy	Person-years	Death from TB or AIDS, N	HR (95% CI)^a^	Person-years	All-cause death, N	HR (95% CI)^a^
0	24,810	288	1.00	25,668	419	1.00
1	16,150	273	1.48 (1.25–1.75)	16,791	381	1.42 (1.23–1.63)
2	9673	228	1.76 (1.45–2.13)	10,323	318	1.70 (1.45–2.00)
3+	16,523	382	1.59 (1.31–1.94)	17,600	547	1.57 (1.33–1.85)

HIV-infected women and women not tested for HIV were combined into the ‘HIV-infected group’ based on similar TB or AIDS mortality rates and proportions of TB or AIDS deaths (Table 1). Additionally, existing studies show that the proportion of HIV-infected adults who were undiagnosed was as high as 80% in the early 2000s [15]. Therefore, individuals not tested for HIV might have actually been HIV-infected

*AIDS* acquired immune deficiency syndrome, *CI* confidence interval, *HR* hazard ratio, *TB* tuberculosis

^a^Hazard ratios were adjusted for age (< 26 and ≥ 26), marital status (never been married, currently married divorced or widowed, and unknown marital status), area of residence (rural, peri-urban, urban, and outside surveillance area) and educational levels (< 1 year of education, 1–6 years of education, 7–12 years of education, ≥ 13 years of education, and unknown educational levels)

**Fig. 4:**
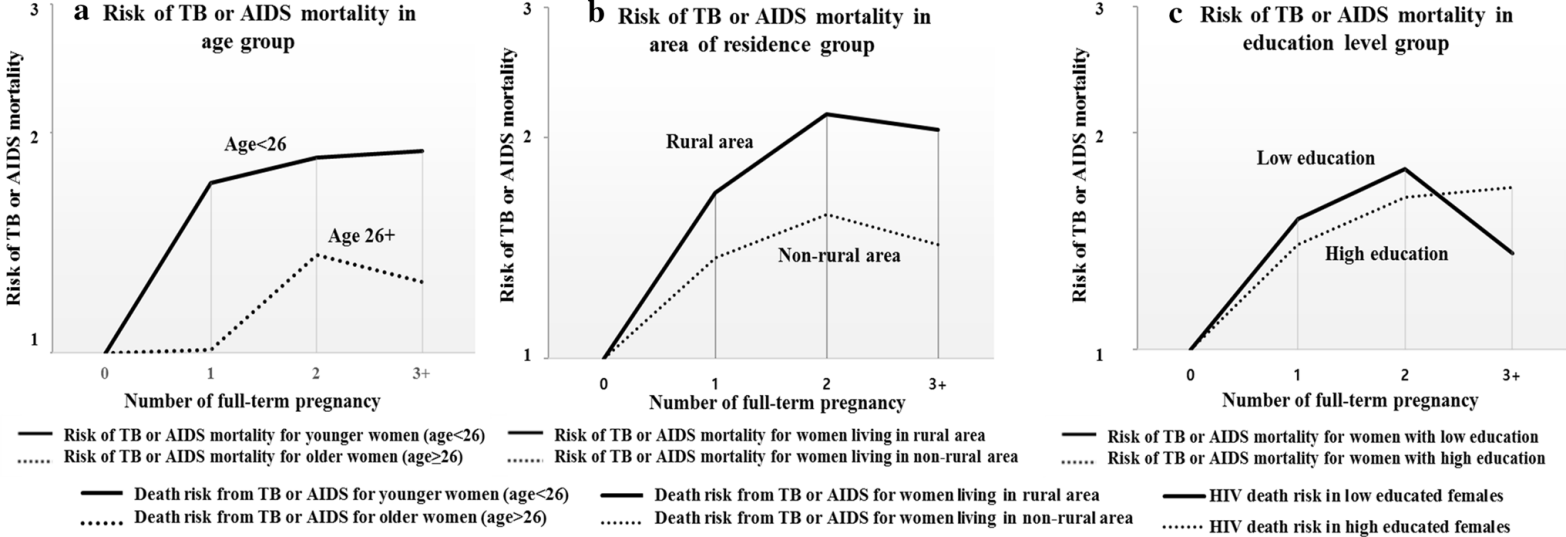
Visualization of the effect of the number of full-term pregnancies on the risk of mortality from TB or AIDS based on **a** age group, **b** area of residence and **c** educational levels. *AIDS* acquired immune deficiency syndrome, *HIV* human immunodeficiency virus, *TB* tuberculosis

In our stratification analysis, younger women (age < 26 years old) and women living in rural areas appeared to have a greater risk of mortality for an increased number of full-term pregnancies (Table 4). In younger women (age < 26), the risk of TB or AIDS mortality increased dramatically as the number of pregnancies increased (HR = 1.71, 1.85, and 1.89 for one, two, and three full-term pregnancies, respectively). For older women (age ≥ 26) with just one full-term pregnancy, the risk of mortality from TB or AIDS was similar (HR = 1.01) to nulliparous women, whereas the risk for younger women with one full-term pregnancy rapidly increased to 1.71-fold (95% CI 1.40–2.10, *P*-heterogeneity = 0.004, and P-interaction < 0.01). The risk of mortality from TB or AIDS for women living in rural areas (HR = 1.68, 2.15 and 2.05 for one, two, and three full-term pregnancies, respectively) was higher than women living in non-rural areas (HR = 1.37, 1.57 and 1.36 for one, two, and three full-term pregnancies, respectively) regardless of the of full-term pregnancies. Among women with higher educational status, the trends in the increase in the risk of TB or AIDS mortality for increased numbers of full-term pregnancies were similar to the patterns observed for all HIV-infected women (a rapid increase until the second pregnancy and a slight increase in the third pregnancy). However, for women with a lower educational status, the following peculiar aspects were observed: the risk of mortality from TB or AIDS increased up to two full-term pregnancies (HR = 1.52 and 1.78 for first and second full-term pregnancies), which was higher than the risk seen in highly educated women (HR = 1.40 and 1.63 for first and second full-term pregnancies), and the risk of death suddenly decreased for more than three full-term pregnancies (HR = 1.36 in poorly educated women vs. HR = 1.68 in highly educated women). Figure 4 visualizes the effect of the number of full-term pregnancies on the risk of mortality from TB or AIDS for the different groups of participants.

**Table 4:** The effect of the number of full-term pregnancies on the risk of TB or AIDS and all-cause mortality in HIV-infected women of reproductive age stratified by age, area of residence, and educational levels

Strata	Full-term pregnancy	Person-years	Death from TB or AIDS, N	HR (95% CI)^a^	Person-years	All-cause death, N	HR (95% CI)^a^
Age < 26	0	21,243	207	1.00	21,977	312	1.00
	1	11,019	174	1.71 (1.40–2.10)^b^	11,383	233	1.52 (1.28–1.81)
	2	3453	62	1.85 (1.38–2.47)	3659	86	1.73 (1.35–2.20)
	3+	1007	19	1.89 (1.16–3.07)	1102	32	2.21 (1.51–3.22)^c^
Age ≥ 26	0	3568	81	1.00	3691	107	1.00
	1	5130	99	1.01 (0.75–1.36)^b^	5408	148	1.13 (0.88–1.45)
	2	6220	166	1.36 (1.04–1.78)	6663	232	1.42 (1.13–1.79)
	3+	15,516	363	1.25 (0.97–1.60)	16,497	515	1.30 (1.05–1.62)^c^
		P-interaction < 0.01			P-interaction = 0.01		
Rural area	0	10,440	101	1.00	10,875	159	1.00
	1	6385	102	1.68 (1.27–2.23)	6601	137	1.44 (1.14–1.82)
	2	3598	77	2.15 (1.55–2.98)	3797	105	1.86 (1.41–2.44)
	3+	7323	154	2.05 (1.45–2.89)	7857	231	1.89 (1.42–2.51)
Non-rural area^d^	0	14,370	187	1.00	14,793	260	1.00
	1	9765	171	1.37 (1.11–1.69)	10,190	244	1.40 (1.17–1.67)
	2	6076	151	1.57 (1.24–1.99)	6526	213	1.62 (1.33–1.98)
	3+	9200	228	1.43 (1.13–1.81)	9743	316	1.45 (1.19–1.78)
		P-interaction = 0.76			P-interaction = 0.89		
Low education level^e^	0	2584	75	1.00	2679	101	1.00
	1	2239	89	1.52 (1.12–2.08)	2315	116	1.45 (1.10–1.89)
	2	2067	101	1.78 (1.31–2.44)	2277	136	1.72 (1.32–2.26)
	3+	7854	238	1.36 (1.01–1.84)	8385	335	1.31 (1.02–1.70)
High education level^6^	0	22,227	213	1.00	22,989	318	1.00
	1	13,911	184	1.40 (1.14–1.71)	14,476	265	1.36 (1.15–1.61)
	2	7607	127	1.63 (1.27–2.08)	8046	182	1.59 (1.30–1.94)
	3+	8669	144	1.68 (1.24–2.14)	9214	212	1.64 (1.31–2.05)
		P-interaction = 0.06			P-interaction = 0.07		

HIV-infected women and women not tested for HIV were combined into the ‘HIV-infected group’ based on similar TB or AIDS mortality rates and proportions of TB or AIDS deaths (Table 1). Additionally, existing studies show that the proportion of HIV-infected adults who were undiagnosed was as high as 80% in the early 2000s [15]. Therefore, individuals not tested for HIV might have actually been HIV-infected

^a^Hazard ratios were adjusted for age (< 26 and ≥ 26), marital status (never been married, currently married divorced or widowed, and unknown marital status), area of residence (rural, peri-urban, urban, and outside surveillance area) and educational levels (< 1 year of education, 1–6 years of education, 7–12 years of education, ≥ 13 years of education, and unknown educational levels)

^b^p-heterogeneity between the two HRs of group [Age < 26] and [Age ≥ 26] = 0.004

^c^p-heterogeneity between the two HRs of group [Age < 26] and [Age ≥ 26] = 0.016

^d^Non-rural areas were defined as peri-ruban, urban, or outside of the surveillance area

^e^Low educational levels were defined as ≤ 6 years of education or unknown educational level and high educational level were defined as > 6 years of education

## Discussion

Our findings are consistent with those from previous studies which have shown that HIV-infected women have considerably greater risk of maternal mortality when compared to their HIV-uninfected counterparts [2]. However, previous studies focused mostly on short-term mortality from pregnancy-related causes during pregnancy or the postpartum period, and not on long-term mortality from major causes of death in HIV-infected populations, such as TB or AIDS.

In our study, we observed that for HIV-infected women, a greater number of pregnancies lead to greater risk of mortality from TB or AIDS, whereas the risk of mortality in HIV-uninfected women was similar regardless of the number of full-term pregnancies. This effect was observed more strongly in women who were younger or living in rural areas than those who were older or living in non-rural areas. Among those with a low educational status, the risk of mortality increased up to two pregnancies, and then rapidly decreased for women with three or more pregnancies to a lower risk than for one full-term pregnancy.

Until now, there have been no studies on the association between the long-term risk of mortality and the number of full-term pregnancies in HIV-infected women. Although there are no previous studies that we can compare or support our results with, our results are biologically plausible. In countries such as the United Kingdom and the United States, pregnancies at younger ages and during multiparous states have been reported to pose physiological health hazards [16–18]. Given the mechanisms of HIV, pregnancy can accelerate the progression of HIV and increase the susceptibility to complications of HIV infection [7, 19]. The susceptibility to infections in HIV-infected pregnant women can be partly explained by an immune shift from cell-mediated immunity (Th1 responses) to humoral immunity (Th2 responses) during pregnancy [20]. Although the purpose of immune alteration is to induce immune tolerance to the fetus, as plasma CD4 cell counts decrease by 75/year on average in the absence of appropriate treatment for HIV-infected women [21], pregnancy may make these women more susceptible to infections [21]. Of course, the standard care for HIV-infected women at pregnancy or postpartum period is to initiate ART at the time of diagnosis and slow down such a rapid decrease in CD4 count. However, in rural areas, such as KwaZulu-Natal, where many women living with HIV still initiate ART in late stages of the disease, low CD counts still poses significant risk, particularly during pregnancy and the postpartum period. Additionally, pregnancy can increase severity of many infectious diseases including TB [7, 19], owing to impairment of pathogen clearance during pregnancy [8]. In fact, TB is the most common opportunistic infection associated with HIV in resource-poor settings [22] and there is higher prevalence of TB infection in HIV-positive pregnant women compared to HIV-uninfected pregnant women [23]. In summary, HIV-infected pregnant women, especially those who become pregnant at younger ages, are burdened with an increased risk of infections as a result of TB and HIV [24].

Despite a considerable decline in total fertility rates since the 1970s, the percentage of women in KwaZulu-Natal, South Africa, a HIV endemic region, giving birth in their teens continues to remain high [25]. In this region, HIV-infected women who are immunocompromised are likely to become more vulnerable to infection, especially owing to numerous pregnancies at younger ages, and are more likely to suffer long-term health consequences and increased risk of all-cause mortality and mortality from TB or HIV.

Although the South African government provides HIV care free of charge in the public sector [26], residing in rural areas still limits geographical accessibility of primary-care clinics to receive routine ART, thus hindering optimal management of HIV. Similarly, younger patients might be more reluctant to take ART than older patients due to social stigma. In fact, younger age is a known factor that has been reported to negatively affect ART adherence in HIV-infected individuals [27]. Such findings help explain our results which showed that younger women (age < 26) and women living in rural area had a greater risk of mortality due to TB or AIDS.

Not surprisingly, lower levels of education are known to negatively impact ART adherence [25]. We had similar findings as the risk of TB or AIDS mortality was increased among HIV-infected women who had one to two full term pregnancies and low levels of education. However, we found that the risk of mortality from TB or AIDS in women with low educational levels decreased for those with more than two pregnancies. Such findings might be explained by the fact that there is a positive relationship between the number of pregnancies and economic levels; the higher socioeconomic status of some makes up for some of the observed disadvantage in health [16]. Additionally, in KwaZulu-Natal, the home of the largest Zulu tribe in South Africa, polygamy is still tolerated and many dowries are paid to the bride for marriage [28]. Therefore, unlike women, husbands may have high socioeconomic and educational status’, and the risk of mortality faced by their spouses may therefore be lowered.

​ This study was subject to several limitations. We had to create a spline plot to infer the effect of the number of full-term pregnancies on all-cause and TB or AIDS mortality in women who experienced four or more full-term pregnancies due to the sparse population of women with high numbers of pregnancies. Further, because most of the deaths in the HIV-infected group (approximately 70%) were attributable to TB or AIDS (Table 1), it was impossible to investigate the effect of the number of full-term pregnancies on the risk of mortality due to non-infectious diseases such as obstetric complications. We were also unable to calculate time interval between HIV contraction and death as we did not have information on when the patients contracted HIV. Pregnancy status during the study period was also self-reported, creating unintentional informational bias. Finally, our data did not investigate the CD4 counts of HIV-infected participants, thus we could not study the progression of HIV and rather had to focus only on the end outcome. To note, we believe that adding more adjustment variables such as initial health status at the beginning of pregnancy, economic percentile of the household, distance to the nearest clinic, health care coverage, access to clean water would further refine our analysis.

Our study used one of the world’s largest population-based HIV cohorts to study the effect of the number of full-term pregnancies on the risk of mortality due to TB or AIDS in a region severely affected by the HIV epidemic. In the ACDIS cohort, the HIV status of participants’ as well as various other factors including sexual behaviors, socioeconomic statuses, and epidemiologic data, were regularly collected for over ten years. Also, each of the three HIV strata used in our study contained more than 2000 study participants. The greatest strength of our study is that it was the first to investigate the influence of the number of pregnancies on the long-term risk of mortality due to HIV-related infections.

In conclusion, our study indicates that higher number of pregnancies in HIV-infected women can substantially increase the long-term risk of mortality, especially due to TB or AIDS. Our results suggest that targeted health policies should be established for women who are planning on becoming or who are currently pregnant in South Africa and other HIV hyperendemic countries, particularly in areas where TB infection is also common. Women who are HIV-infected, especially those with a history of multiple pregnancies, should ensure that they implement measures to control TB and other HIV-related infections before further family planning. ART should be a priority for HIV-infected women who had multiple pregnancies since their early teenage years.
